# Sporoderm Disruption Reshapes the Chemical Characteristics and Enhances the Mitochondrial Protective Activity of *Ganoderma lucidum* Spore Polysaccharides via SIRT1/AMPK Signaling

**DOI:** 10.3390/ijms27114741

**Published:** 2026-05-25

**Authors:** Zhiwei Long, Junzhe Zhang, Longqin Li, Xin Chai, Zipeng Gong, Yang Lu, Hai-Ning Lyu, Shengnan Shen

**Affiliations:** 1 Key Laboratory of Computational Chemistry Based Natural Antitumor Drug Research & Development, Liaoning Province, School of Traditional Chinese Materia Medica, Shenyang Pharmaceutical University, Shenyang 110016, China; 18940833053@163.com; 2 China State Key Laboratory for Quality Ensurance and Sustainable Use of Dao-di Herbs, Artemisinin Research Center, Institute of Chinese Materia Medica, China Academy of Chinese Medical Sciences, Beijing 100700, China; jzzhang@icmm.ac.cn (J.Z.); xchai@icmm.ac.cn (X.C.); 3 State Key Laboratory of Discovery and Utilization of Functional Components in Traditional Chinese Medicine, Engineering Research Center for the Development and Application of Ethnic Medicine and TCM (Ministry of Education), Guizhou Medical University, Guiyang 561113, China; 18375038943@163.com (L.L.); gzp4012607@126.com (Z.G.); 4 Hebei Province Key Laboratory of Study and Exploitation of Chinese Medicine, Chengde Medical University, Chengde 067000, China; luyangmm@cdmc.edu.cn

**Keywords:** *Ganoderma lucidum* spores, polysaccharides, sporoderm disruption, mitochondrial protection, oxidative stress, SIRT1/AMPK signaling

## Abstract

*Ganoderma lucidum* spores are widely consumed natural functional materials; however, the influence of sporoderm disruption on the physicochemical characteristics and biological performance of spore polysaccharides remains insufficiently understood. In this study, polysaccharides extracted from intact spores (GLSP-I) and sporoderm-broken spores (GLSP-SB) were systematically compared to clarify how industrial processing affects their physicochemical properties and cytoprotective activity. Chemical characterization demonstrated that sporoderm disruption increased extraction yield and modified molecular weight distribution, monosaccharide composition, and spectroscopic features without fundamentally altering the polysaccharide backbone. Both polysaccharide fractions alleviated TBHP-induced oxidative injury, whereas GLSP-SB exhibited enhanced cytoprotective efficacy. Quantitative proteomic analysis revealed that GLSP-SB regulated a broader set of proteins associated with mitochondrial organization, oxidative stress response, autophagy, and cellular energy metabolism. Functional assays further showed that GLSP-SB promoted mitochondrial biogenesis, restored ATP production, and maintained mitochondrial morphology under oxidative stress conditions. Mechanistic validation demonstrated activation of the SIRT1/AMPK signaling pathway, indicating that modulation of this pathway contributes to mitochondrial adaptive responses. These findings suggest that sporoderm disruption reshapes polysaccharide characteristics and is associated with enhanced mitochondrial protective activity, providing mechanistic insight into the rational processing and functional utilization of *G. lucidum* spore products.

## 1. Introduction

**Figure 1:**
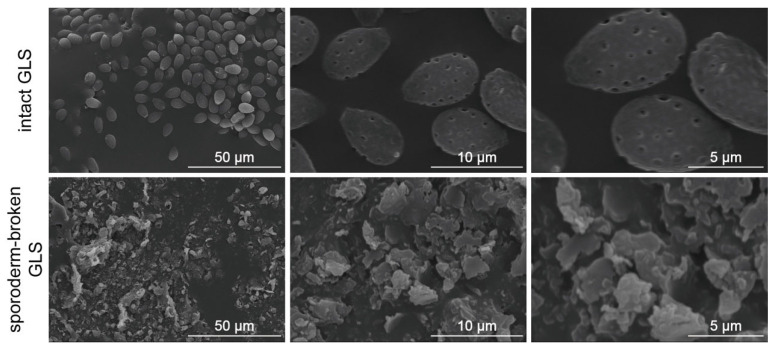
Scanning electron microscopy (SEM) images of intact GLS and sporoderm-broken GLS at different magnifications.

*Ganoderma lucidum* is a well-known medicinal mushroom widely incorporated into functional foods and dietary supplements worldwide. Among its bioresources, *G. lucidum* spores (GLS) have attracted increasing attention owing to their high abundance of bioactive constituents and their recognized status as a homologous material for both food and medicine [1]. Spore-derived preparations exhibit diverse biological activities, including immunomodulatory, antitumor, anti-inflammatory, and metabolic regulatory effects, which have driven the rapid expansion of GLS-based nutraceutical products [2,3,4]. In practical applications, GLS are the most commonly consumed in the powdered form (Figure 1) [5]. However, native spores possess a highly resistant double-layered sporoderm that restricts the release of intracellular constituents and limits bioavailability [6,7,8]. Mechanical sporoderm disruption has therefore become a standard industrial processing strategy to enhance the liberation of polysaccharides and triterpenoids [6,8], and sporoderm-broken products are generally regarded as more effective than intact spores.

Despite widespread industrial adoption, whether sporoderm disruption fundamentally alters the chemical characteristics and biological functions of GLS components remains unclear. Emerging studies indicate that both broken and intact spores can exert comparable biological activities in certain contexts, including immune regulation and prebiotic effects [8,9,10]. Supporting this view, a recent study on GLS oligosaccharides revealed that broken and unbroken spores possess highly similar oligosaccharide compositions and exhibit comparable prebiotic properties, highlighting their shared potential in gut microbiota regulation and immune enhancement [9]. These findings suggest that enhanced bioactivity may not solely result from compound release but may also involve changes in molecular composition or structural organization. In particular, the impact of sporoderm disruption on the structural features and functional properties of spore polysaccharides has not been systematically clarified.

Polysaccharides are considered the principal active constituents of GLS and contribute substantially to their health-promoting properties [1]. While previous studies have mainly focused on immunoregulation, antitumor effects, and antioxidant activities [11,12], increasing evidence highlights their involvement in cellular energy metabolism and mitochondrial homeostasis [13,14]. A recent study demonstrated that GLS polysaccharides (GLSP) ameliorated cognitive deficits in 5×FAD mice by reducing oxidative stress and regulating mitochondrial dynamics via Nrf2 signaling, suggesting a previously underappreciated role of spore polysaccharides in mitochondrial protection [15]. Nevertheless, direct comparisons between polysaccharides derived from intact and sporoderm-broken GLS remain scarce, especially concerning structural variations such as molecular weight distribution and monosaccharide composition, and their possible association with biological performance. Importantly, mitochondrial dysfunction represents a tissue-dependent process, and skeletal muscle, characterized by high energy demand and mitochondrial density, is particularly vulnerable to oxidative stress-induced damage during aging and metabolic decline [16]. These observations suggest that evaluating GLS polysaccharides within a mitochondria-centered muscle model may provide mechanistic insights beyond traditional immunomodulatory investigations.

In the present study, polysaccharides extracted from intact spores (GLSP-I) and sporoderm-broken spores (GLSP-SB) were systematically compared through integrated chemical characterization, functional evaluation, and quantitative proteomics analysis. To interrogate mitochondria-associated protective mechanisms, C2C12 myotubes were employed as an established skeletal muscle model of oxidative stress and mitochondrial dysfunction [17]. By combining structural analysis with mitochondrial functional assessment and mechanistic validation, this study aims to provide new insights into how industrial processing reshapes the polysaccharide profile of GLS and modulates mitochondrial protective activity, thereby offering a scientific basis for the rational processing and utilization of GLS-derived functional products.

## 2. Results

### 2.1. Preparation and Yield of GLSP-I and GLSP-SB

SEM analysis verified successful sporoderm disruption, showing severe destruction of the intact spore wall architecture (Figure 1), providing a structural basis for subsequent polysaccharide extraction. To enable direct comparison between intact and sporoderm-broken spores, identical extraction conditions were applied using a standardized hot water extraction procedure. Crude polysaccharide fractions designated as GLSP-I and GLSP-SB were successfully obtained, with extraction yields of 0.7% and 1.3%, respectively. The increased yield observed for GLSP-SB indicates that mechanical disruption of the resistant sporoderm facilitates the release of intracellular polysaccharides. To further evaluate sample purity and compositional characteristics, total sugar content, uronic acid content, and residual protein levels were determined (Table 1). GLSP-SB exhibited a higher total sugar content (81.6 ± 3.6%) compared with GLSP-I (65.9 ± 2.2%), indicating improved polysaccharide enrichment after sporoderm disruption. Conversely, the uronic acid content decreased from 35.7 ± 1.3% in GLSP-I to 18.5 ± 0.7% in GLSP-SB, suggesting preferential extraction of neutral polysaccharide fractions. The total sugar and uronic acid contents reflect overall carbohydrate abundance and the relative proportion of acidic polysaccharide residues, respectively, providing complementary compositional information.

### 2.2. Molecular Weight Distribution

**Figure 2:**
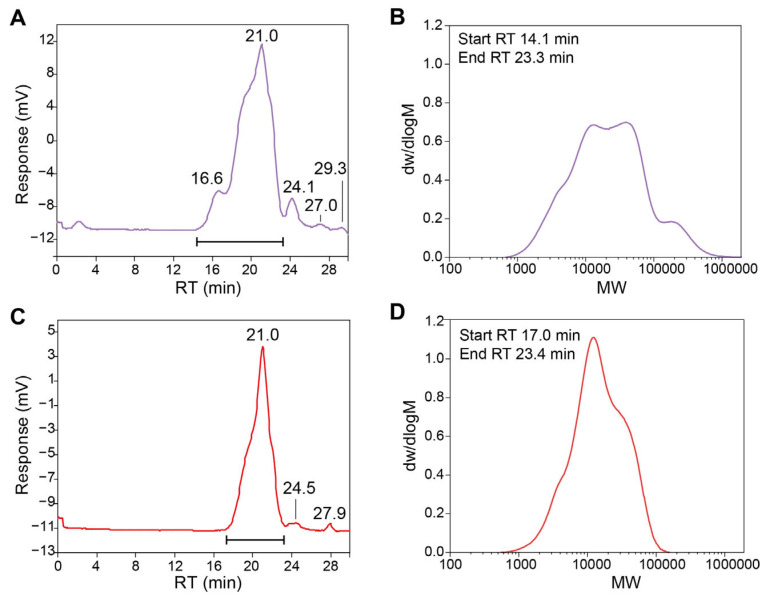
Molecular weight distribution profiles of GSLP-I and GSLP-SB. (**A**) GPC chromatogram of GSLP-I showing the molecular weight distribution across the full elution range. (**B**) Enlarged view of the chromatogram of GSLP-I highlighting the molecular weight distribution of GSLP-I within the specified time windows in (**A**). (**C**) GPC chromatogram of GSLP-SB showing the molecular weight distribution across the full elution range. (**D**) Enlarged view of the chromatogram of GSLP-SB, highlighting the molecular weight distribution of GSLP-SB within the specified time windows in (**C**).

The molecular weight distribution of GLSP-I and GLSP-SB was analyzed by gel permeation chromatography (GPC), and the corresponding chromatograms and calculated parameters are presented in Figure 2 and Table 2. GLSP-I exhibited a complex chromatographic profile composed of multiple elution peaks, indicating the presence of polysaccharide fractions with markedly different molecular sizes. Five major components were detected with retention times of 16.6, 21.0, 24.1, 27.0, and 29.3 min, corresponding to weight-average molecular weights (Mw) ranging from 6.01 × 10^6^ Da to 1.57 × 10^3^ Da. The dominant high-molecular-weight fraction observed at earlier retention time indicates the presence of abundant high-molecular-weight polysaccharide populations together with several medium- and low-molecular-weight components, reflecting pronounced structural heterogeneity. In contrast, GLSP-SB displayed a simplified molecular weight distribution characterized by three primary fractions eluting at 21.0, 24.5, and 27.9 min. The Mw values of these fractions were 4.29 × 10^5^ Da, 4.65 × 10^4^ Da, and 5.16 × 10^3^ Da, respectively. Compared with GLSP-I, the disappearance of the ultra-high-molecular-weight component and the reduction in peak number indicate that sporoderm disruption markedly altered the polysaccharide size distribution. The chromatogram of GLSP-SB showed a narrower and more concentrated peak pattern, suggesting improved molecular homogeneity. These results imply that mechanical disruption of the sporoderm not only enhances polysaccharide release but may reflect fragmentation and/or preferential extraction of specific polysaccharide populations, leading to a more uniform molecular architecture.

### 2.3. Monosaccharide Composition

**Figure 3:**
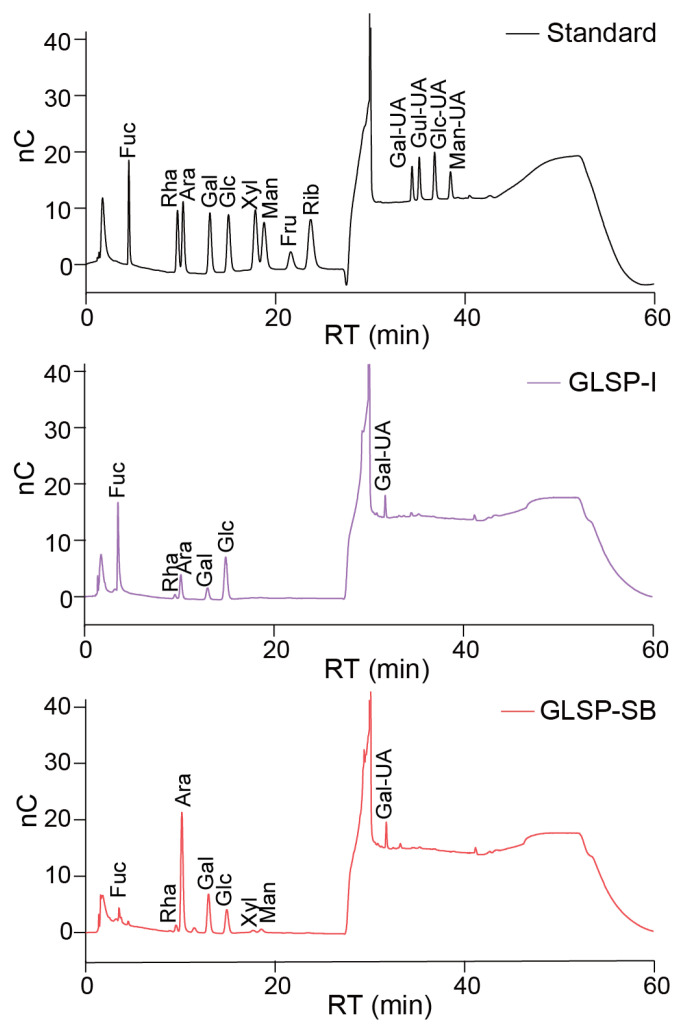
Comparison of the monosaccharide composition of GLSP-I and GLSP-SB.

The monosaccharide compositions of GLSP-I and GLSP-SB were determined after acid hydrolysis followed by ion chromatography analysis, and the results are summarized in Figure 3 and Table 3. Both polysaccharide fractions consisted of neutral sugars and uronic acids but exhibited distinct quantitative differences. GLSP-I was mainly composed of glucose and galacturonic acid, whereas GLSP-SB showed a pronounced enrichment of arabinose and galactose, accompanied by a marked reduction in glucose and uronic acid content. Additionally, minor amounts of fucose, xylose, and mannose were detected only in GLSP-SB. Overall, sporoderm disruption altered the relative monosaccharide distribution while preserving the general polysaccharide framework.

### 2.4. Spectroscopic Characterization

**Figure 4:**
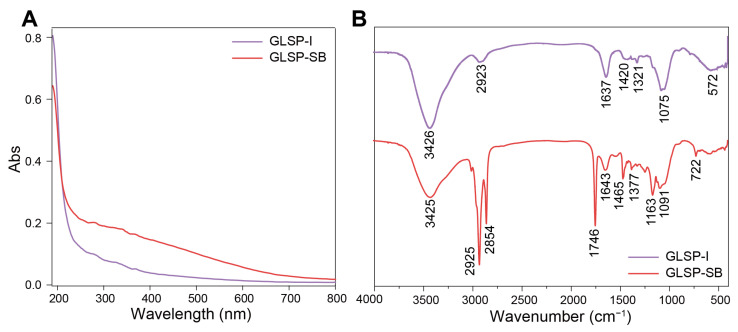
Spectroscopic characterization of GLSP-I and GLSP-SB. (**A**) UV-Vis absorption spectra of polysaccharides recorded from 190 to 800 nm. (**B**) FT-IR spectra of GLSP-I and GLSP-SB obtained in the range of 4000−400 cm^−1^.

The UV-Vis absorption and FT-IR spectra of GLSP-I and GLSP-SB are presented in Figure 4 to further characterize their purity and structural features. As shown in Figure 4A, both polysaccharide fractions exhibited strong absorption in the ultraviolet region below 220 nm, typical of polysaccharides. No obvious absorption peaks were observed at 260 or 280 nm, indicating the absence of detectable nucleic acid or protein contamination and supporting the high purity of the extracted polysaccharides. Compared with GLSP-I, GLSP-SB displayed slightly higher absorbance intensity, consistent with compositional differences between the two fractions.

The FT-IR spectra (Figure 4B) revealed typical polysaccharide structural features in both samples. A broad absorption band around 3425–3426 cm^−1^ corresponded to O–H stretching vibrations, while peaks near 2923–2925 cm^−1^ were attributed to C–H stretching of sugar residues. The absorption bands observed at approximately 1637–1643 cm^−1^ were associated with bound water and carbonyl groups, indicating the presence of uronic acid components, which is consistent with monosaccharide composition analysis. Notably, GLSP-SB exhibited additional or intensified absorption signals, particularly the band near 1746 cm^−1^, indicating possible alterations in acidic functional groups after sporoderm disruption. Strong signals within the 1200–1000 cm^−1^ region were assigned to C–O–C and glycosidic bond vibrations typical of pyranose polysaccharides [18]. Subtle variations in this region further imply alterations in glycosidic linkage environments and branching structures. Overall, spectroscopic analyses confirmed that both fractions share common polysaccharide backbones, while sporoderm disruption induced detectable compositional and physicochemical differences, in agreement with the molecular weight distribution and monosaccharide composition results. Notably, the absence of characteristic protein-associated amide I (~1650 cm^−1^) and amide II (~1540 cm^−1^) bands, together with the low residual protein content determined by BCA assay, further verified the satisfactory purity of the obtained polysaccharide fractions.

### 2.5. GLSP-SB Exhibits Enhanced Cytoprotective Effects Against Oxidative Stress

**Figure 5:**
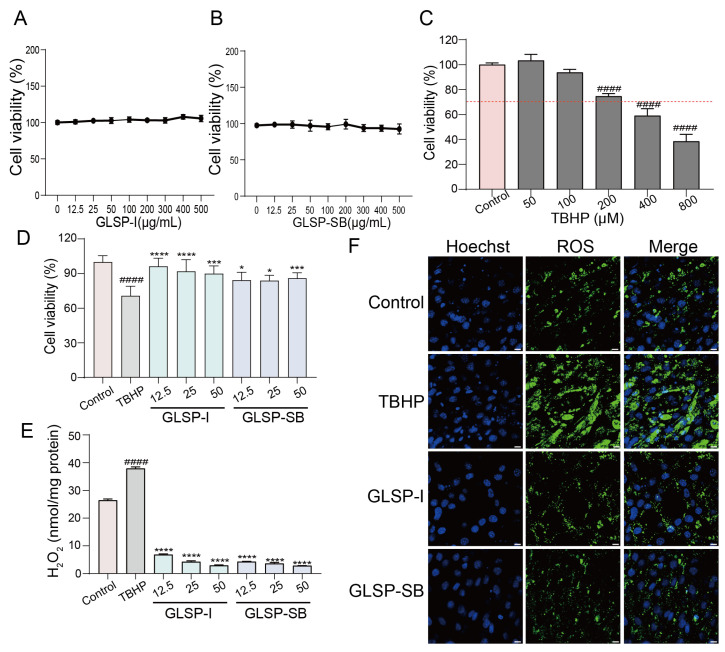
Cytoprotective effects of GLSP-I and GLSP-SB against TBHP-induced oxidative stress in C2C12 myotubes. (**A**,**B**) Effects of GLSP-I and GLSP-SB on C2C12 myotube cell viability after 24 h treatment under different concentrations (0–500 μg/mL). (**C**) Optimization of oxidative stress conditions. Cells were exposed to TBHP (50–800 μM) for 24 h, and 200 μM was selected as it reduced cell viability to approximately 80% while maintaining experimental stability. (**D**) Cell viability following treatment with GLSP-I or GLSP-SB at indicated concentrations under TBHP-induced oxidative stress. (**E**) Intracellular H_2_O_2_ levels after polysaccharide treatment. (**F**) Representative fluorescence images of intracellular ROS levels detected using DCFH-DA staining. Nuclei were stained with Hoechst (blue), and ROS signals are shown in green. Bar = 10 μm. Data are presented as mean ± SD (*n* = 3). #### *p* < 0.0001 vs. control group; * *p* < 0.05, *** *p* < 0.001, **** *p* < 0.0001 vs. TBHP group.

GLSP-I and GLSP-SB showed no obvious cytotoxicity at concentrations of 0–1000 μg/mL with a cell survival rate exceeding 90% (Figure 5A,B). To evaluate the protective activity, a TBHP-induced oxidative stress model was first optimized in C2C12 myotubes. As shown in Figure 5C, exposure to increasing TBHP concentrations (50–800 μM) resulted in a progressive decrease in cell viability, and 200 μM TBHP was selected for subsequent experiments as it produced stable oxidative injury while maintaining sufficient viable cells for functional assessment. Under these conditions, TBHP treatment markedly reduced cell viability compared with the control group (Figure 5D), confirming successful establishment of the oxidative damage model [19]. Pretreatment with both GLSP-I and GLSP-SB significantly improved cell survival in a concentration-dependent manner. Notably, GLSP-SB consistently exhibited higher protective efficacy than GLSP-I at comparable concentrations, suggesting enhanced bioactivity following sporoderm disruption. Consistent with the cell viability results, intracellular H_2_O_2_ levels were significantly elevated after TBHP exposure (Figure 5E). Treatment with both polysaccharide fractions effectively mitigated oxidative damage accumulation, with GLSP-SB exhibiting superior pharmacological activity. Fluorescence imaging further demonstrated that TBHP stimulation induced strong intracellular ROS generation, whereas treatment with GLSP-I attenuated ROS signals and GLSP-SB restored fluorescence intensity closer to basal levels (Figure 5F). These findings demonstrate that GLSP fractions effectively alleviate oxidative stress-induced injury, and that sporoderm disruption enhances cytoprotective efficiency, likely associated with altered polysaccharide characteristics.

### 2.6. Proteomics Analysis Reveals Broader Regulation by GLSP-SB

**Figure 6:**
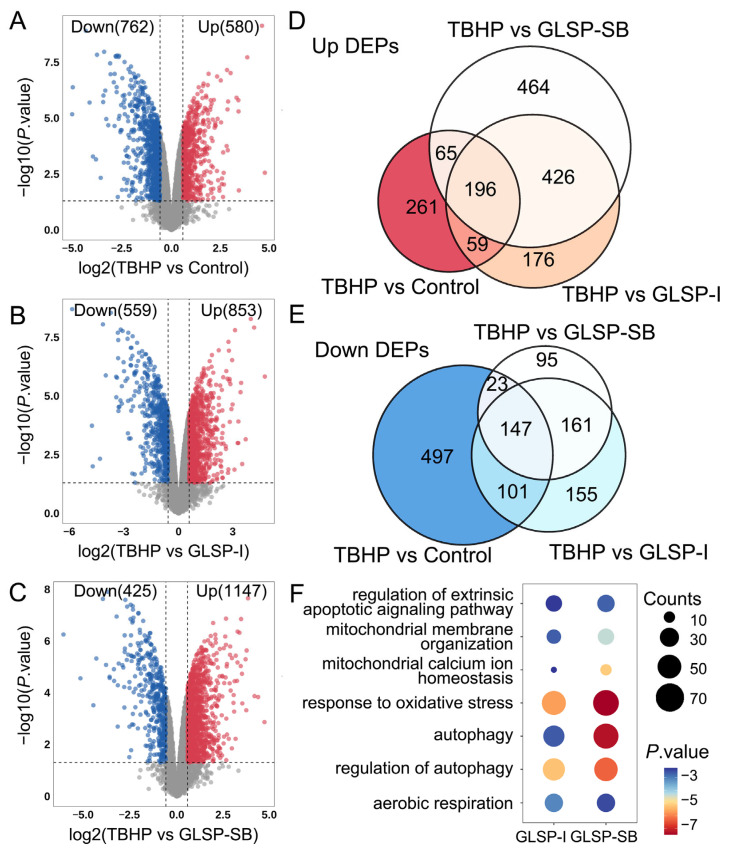
Proteomics profiling reveals differential regulatory effects of GLSP-I and GLSP-SB on oxidative stress-induced proteome remodeling. Volcano plots showing differentially expressed proteins (DEPs) in TBHP vs. control (**A**) Volcano plot of TBHP vs. control: severe oxidative stress induces 580 upregulated (red) and 762 downregulated (blue) DEPs. (**B**) Volcano plot of TBHP vs. GLSP-I: partial proteomic rescue with 853 upregulated and 559 downregulated DEPs vs. TBHP. (**C**). Volcano plot of TBHP vs. GLSP-SB: enhanced rescue with 1147 upregulated and 425 downregulated DEPs vs. TBHP. Color coding: Red, significantly upregulated proteins; Blue, significantly downregulated proteins; Gray, non-significant proteins. (**D**) and downregulated (**E**) DEPs among different comparison groups. (**F**) GO enrichment analysis of biological processes associated with DEPs regulated by GLSP-I and GLSP-SB, highlighting pathways related to oxidative stress response, mitochondrial function, apoptosis, autophagy, and aerobic respiration.

Given the enhanced cytoprotective efficacy of GLSP-SB, quantitative proteomic analysis was performed to elucidate the underlying molecular mechanisms (Dataset S1). As shown in Figure 6A, TBHP exposure induced extensive proteomic remodeling compared with the control group, resulting in 580 upregulated and 762 downregulated proteins, indicating severe disruption of cellular homeostasis under oxidative stress conditions. Treatment with GLSP-I partially reversed TBHP-induced alterations, with 853 upregulated and 559 downregulated proteins identified relative to the TBHP group (Figure 6B). Notably, GLSP-SB treatment induced a more pronounced proteomic reprogramming, characterized by 1147 upregulated and 425 downregulated proteins (Figure 6C), indicating broader proteomic modulation against oxidative stress-mediated cellular injury. Venn diagram analysis further revealed substantial overlap between proteins altered by TBHP exposure and those modulated by polysaccharide treatments (Figure 6D,E). Both GLSP-I and GLSP-SB reversed a considerable proportion of TBHP-induced differential proteins; however, GLSP-SB affected a larger subset of shared targets associated with stress adaptation and cellular recovery. These findings indicate that although both polysaccharide fractions exert protective effects, sporoderm disruption enhances the breadth and efficiency of proteomic regulation. GO enrichment analysis demonstrated that the differentially expressed proteins regulated by both polysaccharides were predominantly involved in biological processes related to mitochondrial function and oxidative stress responses, including mitochondrial membrane organization, mitochondrial calcium ion homeostasis, aerobic respiration, autophagy, and regulation of apoptosis (Figure 6F). Importantly, GLSP-SB showed stronger enrichment in pathways associated with oxidative stress response and autophagy regulation, suggesting improved maintenance of mitochondrial quality control and energy metabolism. Although GLSP-I and GLSP-SB exhibited comparable cytoprotective effects at the phenotypic level, GLSP-SB demonstrated broader and more coordinated regulation of mitochondrial stress-responsive proteins. These results suggested mitochondrial regulation as a potential central mechanism underlying GLSP-SB activity, providing the rationale for focusing subsequent mechanistic studies on GLSP-SB.

### 2.7. GLSP-SB Restores Mitochondrial Function Under Oxidative Stress

**Figure 7:**
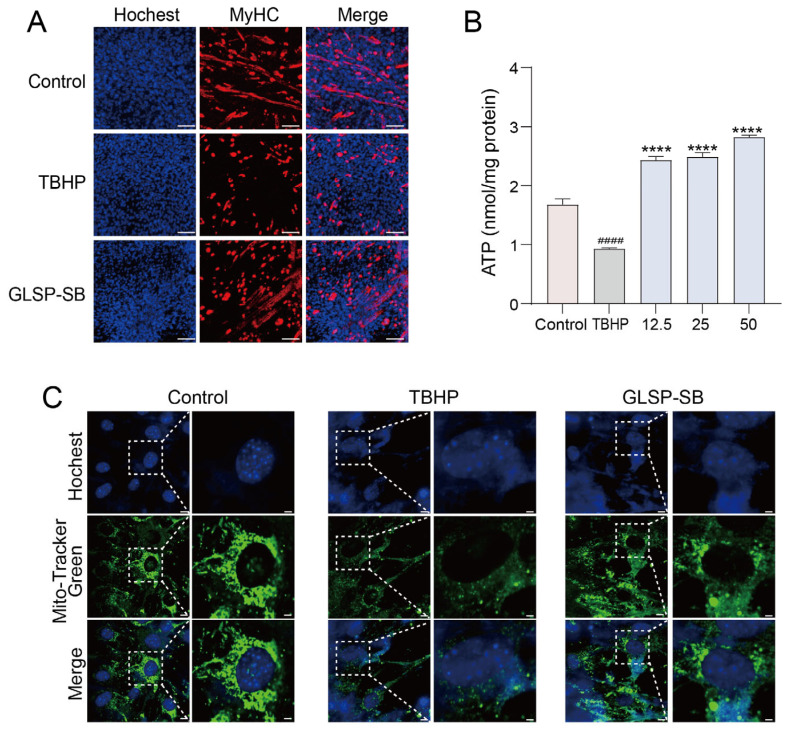
GLSP-SB restores mitochondrial function in TBHP-induced oxidative stress model. (**A**) Immunostaining of MyHC (myosin heavy chain) in TBHP-treated C2C12 myotubes. MyHC is stained in red (Alexa Fluor 647); Nuclei were stained with DAPI (blue). Bar = 100 μm. (**B**) Intracellular ATP levels measured after GLSP-SB treatment at different concentrations. (**C**) Representative fluorescence images showing mitochondrial morphology visualized by Mito-Tracker Green staining. Bright green filamentous structures indicate healthy, interconnected mitochondria. Nuclei were stained with Hoechst (blue). TBHP treatment induced mitochondrial fragmentation and loss of network integrity, whereas GLSP-SB markedly preserved mitochondrial structure. Enlarged regions are indicated by dashed boxes. Bar = 10 μm. Data are presented as mean ± SD. #### *p* < 0.0001 vs. control group; **** *p* < 0.0001 vs. TBHP group.

The proteomics analysis suggested that GLSP-SB predominantly regulated pathways associated with mitochondrial organization, aerobic respiration, and oxidative stress responses. To functionally validate these findings, mitochondrial morphology and bioenergetic parameters were further evaluated in the TBHP-induced oxidative injury model [20]. As shown in Figure 7A, control cells exhibited intact myofibrils with thick myofibrillar structure distributed throughout the cytoplasm. Exposure to TBHP resulted in pronounced myofibrillar fragmentation and disruption of filament continuity, indicating severe skeletal muscle damage. In contrast, GLSP-SB treatment markedly preserved myofibrillar morphology, maintaining a more integrated and filamentous architecture, suggesting protection against oxidative stress-induced myofibrillar disruption. Given that skeletal muscle function is highly dependent on mitochondrial ATP supply, we next assessed cellular ATP levels to evaluate mitochondrial bioenergetic capacity (Figure 7B). TBHP stimulation significantly reduced ATP levels compared with the control group, reflecting mitochondrial dysfunction. Notably, GLSP-SB treatment dose-dependently restored ATP generation, with higher concentrations nearly reversing TBHP-induced mitochondrial dysfunction, thereby demonstrating improved mitochondrial bioenergetic capacity. Finally, we employed fluorescence imaging to directly visualize mitochondrial structural integrity (Figure 7C). Control cells exhibited an interconnected mitochondrial network with elongated filamentous structures distributed throughout the cytoplasm. Exposure to TBHP resulted in pronounced mitochondrial fragmentation and disruption of network continuity, indicating severe mitochondrial damage. In contrast, GLSP-SB treatment markedly preserved mitochondrial morphology, maintaining a more integrated and reticular structure, suggesting protection against oxidative stress-induced mitochondrial dysfunction. These results demonstrate that GLSP-SB effectively alleviates oxidative stress-induced mitochondrial dysfunction by preserving mitochondrial structure, sustaining energy production, and maintaining membrane potential. These functional findings validate the proteomic prediction that mitochondrial function regulation represents a central mechanism underlying the cytoprotective activity of GLSP-SB.

### 2.8. Validation of SIRT1/AMPK-Mediated Mitochondrial Regulatory Pathway

**Figure 8:**
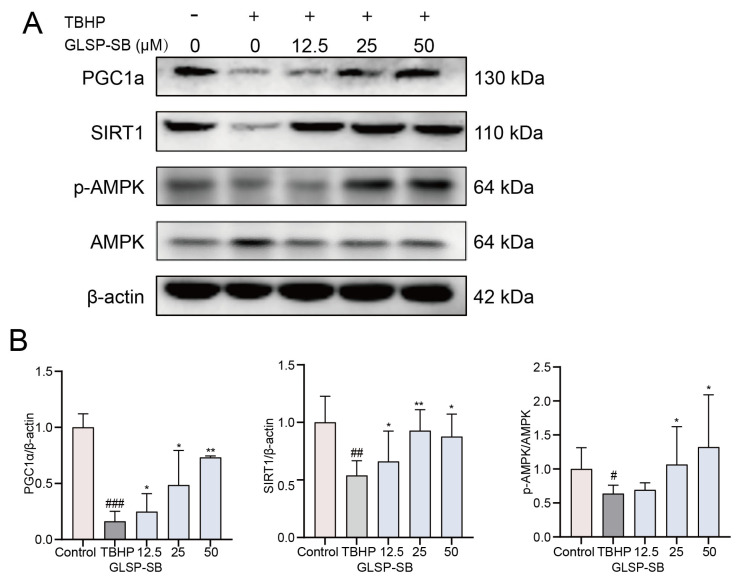
GLSP-SB activates the SIRT1/AMPK–PGC1α signaling pathway under oxidative stress conditions. (**A**) Representative Western blot analysis showing the expression levels of PGC1α, SIRT1, phosphorylated AMPK (p-AMPK), total AMPK, and β-actin. Lane labels: "-" indicates control; "+" indicates TBHP-treated (200 μM, 24 h). (**B**) Quantification of expression of PGC1α/β-actin, SIRT1/β-actin, and p-AMPK/AMPK expression ratio. Data are expressed as mean ± SD. # *p* < 0.05, ## *p* < 0.01, ### *p* < 0.0001 vs. control group; * *p* < 0.05, ** *p* < 0.01 vs. TBHP group.

Proteomic analysis identified mitochondrial regulation as a central process modulated by GLSP-SB. To further validate the molecular mechanism underlying these effects, key signaling proteins involved in mitochondrial regulation were examined by Western blot analysis. Previous studies have established that AMPK and SIRT1 serve as critical molecular switches governing skeletal muscle energy metabolism, while PGC1α functions as a master regulator promoting mitochondrial biogenesis. Activation of this signaling axis is indicative of enhanced mitochondrial biogenesis and metabolic adaptation [21]. As shown in Figure 8, TBHP exposure markedly decreased the expression of mitochondrial biogenesis-related regulators, including SIRT1 and PGC1α, accompanied by reduced phosphorylation of AMPK, indicating suppression of mitochondrial adaptive signaling under oxidative stress conditions. In contrast, GLSP-SB treatment significantly reversed these alterations in a dose-dependent manner. Specifically, GLSP-SB progressively restored SIRT1 protein expression compared with the TBHP group. Consistently, the downstream transcriptional coactivator PGC1α, a central regulator of mitochondrial biogenesis and oxidative metabolism, was also markedly upregulated following GLSP-SB administration. Moreover, GLSP-SB enhanced AMPK phosphorylation while total AMPK expression remained relatively unchanged, suggesting the activation effect of AMPK. These findings demonstrate that GLSP-SB alleviates oxidative stress-induced mitochondrial dysfunction through activation of the SIRT1/AMPK–PGC1α signaling axis. Together with proteomic profiling and mitochondrial functional validation, these results establish a mechanistic framework in which GLSP-SB preserves mitochondrial homeostasis through activation of the SIRT1/AMPK–PGC1α signaling axis, thereby conferring resistance to oxidative stress-induced cellular injury.

## 3. Discussion

Sporoderm disruption has been widely applied to improve the utilization efficiency of GLS, yet the molecular basis underlying its enhanced bioactivity remains insufficiently clarified [8]. In the present study, integrated structural analyses demonstrated that sporoderm breaking not only increased polysaccharide yield but also reshaped key structural features, including molecular weight distribution and monosaccharide composition. Compared with polysaccharides from intact spores, GLSP-SB exhibited reduced molecular heterogeneity and enrichment of arabinose- and galactose-containing components. Previous studies have suggested that GLSP with moderate molecular weights (10^4^–10^5^ Da) display improved bioaccessibility and biological activity, whereas ultra-high-molecular-weight fractions may exhibit limited functional availability [22]. The disappearance of the ultra-high Mw fraction and enrichment of medium-Mw populations observed here are therefore consistent with reported structure–function tendencies. Similarly, Fang et al. reported enhanced anticancer activity associated with increased arabinose and galactose content following sporoderm removal [23], supporting the notion that processing-induced compositional redistribution contributes to functional improvement. The higher total sugar content and reduced uronic acid proportion in GLSP-SB further indicate preferential enrichment of neutral arabinogalactan-rich polysaccharides. Importantly, sporoderm disruption likely involves both enhanced intracellular release and selective extraction rather than simple degradation, although their relative contributions cannot be distinguished within the present dataset. Together with previous studies on mushroom polysaccharides linking optimized molecular size and arabinogalactan enrichment to improved antioxidant and cytoprotective effects [22,24], our findings suggest that mechanical processing itself may act as a structural optimization step that reshapes polysaccharide populations toward more bioactive configurations.

While *Ganoderma* polysaccharides are frequently investigated for immunomodulatory effects, increasing evidence indicates that mitochondrial dysfunction and oxidative stress are central drivers of aging-related decline [16], particularly in skeletal muscle, a tissue characterized by high mitochondrial density and energy demand [25]. On this basis, differentiated C2C12 myotubes were employed to investigate mitochondria-centered cytoprotective mechanisms [26]. Functionally, both GLSP fractions alleviated TBHP-induced oxidative injury, whereas GLSP-SB exhibited markedly superior protective efficacy, as quantitatively supported by a substantially reduced EC_50_ value. Quantitative proteomics further revealed that the enhanced protection extended beyond direct ROS scavenging and involved coordinated regulation of aerobic respiration, mitochondrial organization, autophagy, and cellular stress adaptation. Similar mitochondria-targeted regulatory effects have been reported for polysaccharides from medicinal fungi and plants, which promote cellular resilience through metabolic reprogramming rather than simple ROS scavenging [15,27,28]. Compared with these studies, the present work uniquely links industrial processing (sporoderm disruption) with downstream mitochondrial functional remodeling, thereby connecting natural product processing chemistry with cellular pharmacology at the systems level.

Mechanistic validation demonstrated that GLSP-SB preserves mitochondrial homeostasis by restoring mitochondrial morphology, energy production, and membrane potential, accompanied by activation of the SIRT1/AMPK–PGC1α signaling axis. This pathway represents a conserved metabolic regulatory network regulating mitochondrial biogenesis and adaptive responses to energetic stress and has been increasingly recognized as a key target of natural product-mediated anti-aging interventions [21,27,28]. Although previous studies associated *G. lucidum* polysaccharides with AMPK activation in intestinal or metabolic models [27], the present study provides evidence that sporoderm disruption enhances activation of this axis specifically in muscle cells under oxidative stress conditions. Collectively, these findings support a working model in which processing-induced modification of polysaccharide characteristics translates into improved mitochondrial adaptability and cellular stress resistance. Nevertheless, the present work remains limited to an in vitro evaluation. Considering the complex in vivo metabolism of polysaccharides, including gastrointestinal digestion, gut microbiota transformation, and potentially limited systemic absorption, future studies should prioritize validation in animal models of aging or oxidative stress, together with investigations of bioavailability, metabolic fate, and tissue-specific responses to better clarify physiological relevance and translational potential. In addition, although GLS contain multiple classes of bioactive constituents, the present study specifically focuses on water-soluble polysaccharides as representative macromolecular effectors influenced by sporoderm disruption. Comprehensive investigations integrating polysaccharides with other chemical components will be valuable for achieving a more holistic understanding of how industrial processing reshapes the overall bioactivity of GLS.

## 4. Materials and Methods

### 4.1. Materials

Dried *Ganoderma lucidum* spores were obtained from the Anguo Herbal Medicine Market (Hebei, China). Sporoderm disruption was performed using an ultrafine grinding system (Yantai Huibao Equipment Manufacturing Co., Ltd., Yantai, China). Monosaccharide reference standards, including arabinose (Ara), galactose (Gal), glucose (Glc), mannose (Man), fructose (Fru), rhamnose (Rha), ribose (Rib), and xylose (Xyl), were supplied by Shanghai Yuanye Biotechnology Co., Ltd. (Shanghai, China). Trypsin was purchased from Promega (Madison, WI, USA). The Pierce™ Quantitative Colorimetric Peptide Assay Kit and C18 desalting columns used for LC–MS/MS analysis were obtained from Thermo Fisher Scientific (Waltham, MA, USA). All other reagents were of analytical grade unless otherwise specified.

### 4.2. Scanning Electron Microscopy (SEM)

The surface morphology of intact and sporoderm-broken GLS was observed using a scanning electron microscope (Hitachi S-3400N, Tokyo, Japan). Samples were mounted on aluminum stubs with conductive carbon tape, sputter-coated with a gold–palladium layer, and examined under vacuum at an accelerating voltage of 10 kV. Representative images were recorded at different magnifications.

### 4.3. Extraction of Ganoderma lucidum Spore Polysaccharides (GLSP)

Powdered intact spores and sporoderm-broken spores (20 g each) were subjected to hot water extraction. Samples were refluxed with 200 mL of distilled water for 2 h, and the extraction procedure was repeated twice to ensure sufficient polysaccharide recovery. The pooled aqueous extracts were filtered and concentrated under reduced pressure. Polysaccharides were subsequently precipitated by the addition of ethanol to a final concentration of 70% (*v*/*v*) and maintained at 4 °C overnight. The precipitated materials were collected by centrifugation at 4000 rpm for 10 min and washed several times with absolute ethanol to remove low-molecular-weight impurities. The resulting residues were freeze-dried to obtain crude polysaccharides derived from intact spores and sporoderm-broken spores, designated as GLSP-I and GLSP-SB, respectively. All extractions were independently performed in triplicate.

### 4.4. UV-Vis Absorption Spectra and FT-IR Spectroscopic Analysis

UV-Vis absorption spectra of GLSP-I and GLSP-SB were recorded using a TU-1900 spectrophotometer (PGeneral, Beijing, China) within the wavelength range of 190–800 nm. The analysis was conducted to assess the presence of potential impurities such as proteins and nucleic acids in the polysaccharide preparations. Fourier-transform infrared (FT-IR) spectroscopy was performed to characterize the functional groups of the polysaccharide samples. Freeze-dried polysaccharides were thoroughly blended with spectroscopic-grade KBr powder and compressed into transparent pellets. Infrared spectra were collected using a Nicolet iS5 FT-IR spectrometer (Thermo Fisher Scientific, Waltham, MA, USA) over the spectral range of 4000–400 cm^−1^ with a resolution of 4 cm^−1^.

### 4.5. Determination of Total Sugar, Uronic Acid, and Residual Protein Contents

Total sugar content was determined by the phenol–sulfuric acid method using glucose as the standard [29]. Uronic acid content was measured by the carbazole–sulfuric acid method using galacturonic acid as the standard [30]. Residual protein content was quantified using the BCA Protein Assay Kit (Beyotime Biotechnology, P0012S, Shanghai, China), following the manufacturer’s instructions. All measurements were performed in triplicate.

### 4.6. Molecular Weight Distribution Analysis

The molecular weight profiles of polysaccharides were analyzed using gel permeation chromatography (GPC). Each sample (5 mg) was dissolved in 1 mL of distilled water and allowed to hydrate completely for 1 h. Prior to injection, the solutions were filtered through a 0.22 μm membrane filter. Chromatographic separation was carried out on a Waters e2695 system (Waters Corp., Milford, MA, USA) equipped with a refractive index detector. A PL aquagel-OH MIXED-M column (7.5 × 300 mm, Agilent Technologies, Santa Clara, CA, USA) was employed at 40 °C using distilled water as the eluent at a flow rate of 1.0 mL/min. A calibration curve was established using linear polyethylene glycol (PEG) standards, and the apparent molecular weight parameters and distribution characteristics of the polysaccharide samples were calculated according to their retention behavior.

### 4.7. Monosaccharide Composition Analysis

Monosaccharide composition was determined by acid hydrolysis (2 M TFA, 121 °C, 2 h) followed by HPAEC-PAD (Dionex CarboPac PA20 column, 150 × 3.0 mm, 10 μm; Thermo ICS-5000+ with PAD, Thermo Fisher Scientific, Waltham, MA, USA). Samples were hydrolyzed in sealed tubes, dried under nitrogen, washed with methanol (3 × 1 mL), and re-dissolved in water. The mobile phase consisted of A (H_2_O), B (0.1 M NaOH), and C (0.1 M NaOH + 0.2 M NaAc), with gradient elution: 0 min 95:5:0; 26 min 85:5:10; 42.1 min 60:0:40; 52 min 60:40:0; 52.1 min 95:5:0; and 60 min 95:5:0 (A/B/C, *v*/*v*/*v*). Flow rate of 0.5 mL/min; injection: 5 μL; column temperature of 30 °C. Standard curves (8 levels, 0.4–60 μg/mL, Sigma-Aldrich standards, Sigma Chemical Co., St. Louis, MO, USA) showed r^2^ ≥ 0.99. The matrix-induced retention time shift for galacturonic acid (~14.2 min in hydrolysates vs. ~15.2 min in standards) was compensated for by matched calibration curves. Quantification was performed using Chromeleon 7.2 CDS software by comparing retention times and peak areas with authentic standards. Each sample was analyzed in triplicate injections to ensure analytical reproducibility.

### 4.8. Cell Culture

C2C12 myoblasts (American Type Culture Collection, ATCC) were grown in high-glucose Dulbecco’s Modified Eagle Medium (DMEM), supplemented with 10% heat-inactivated fetal bovine serum (FBS) and 1% penicillin/streptomycin (P/S), in a humidified incubator at 37 °C with 5% CO_2_. The cells reached 70–80% confluence and were incubated with differentiation medium (DMEM supplemented with 2% heat-inactivated horse serum and 1% P/S) for 4 days. Tert-butyl hydroperoxide (TBHP) (200 μM) was used to treat the fully differentiated myotubes, with or without the existence of GLSP, as indicated for 24 h. DMSO was treated as a vehicle control.

### 4.9. Cell Viability Assay

Cell viability was assessed by the Cell Counting Kit-8 (CCK-8 assay; MeilunBio, Dalian, China). C2C12 myoblasts were seeded into 96-well plates at a density of 5 × 10^4^ cells/well and subsequently induced to differentiate. The myotubes were treated with different concentrations of GLSP for 24 h with or without 200 μM TBHP after being fully differentiated. Next, the cells were incubated for another 30 min after adding 10 μL enhanced CCK-8 solution to each well. Absorbance was measured at 450 nm using a multimode microplate reader (EnVision 2104, PerkinElmer, Waltham, MA, USA).

### 4.10. Intracellular ROS via DCFH-DA Staining

The intracellular ROS level was measured using DCFH-DA fluorescent probing (Beyotime Biotechnology, Shanghai, China) after the specified treatments. After drug treatment or intervention, C2C12 myotubes were loaded with 10 μM DCFH-DA for the last 30 min of incubation at 37 °C in the dark. Excess probe was removed prior to imaging, and fluorescence signals were captured using a confocal fluorescence microscope (Leica TCS SP8 SR, Wetzlar, Germany) and analyzed with ImageJ software (version 1.53t, Java 1.8.0, NIH, Bethesda, MD, USA). ROS generation was quantified by measuring relative fluorescence intensity, which indicated the intracellular oxidative status.

### 4.11. Determination of H_2_O_2_ Level

Intracellular H_2_O_2_ levels were quantified using the Hydrogen Peroxide Assay Kit (Beyotime Biotechnology, Shanghai, China) as previously described [26]. In brief, the harvested cells were lysed and centrifuged at 12,000× *g* for 5 min. Next, the cell lysate and test solution were incubated for 30 min at room temperature. Absorbance was measured at 560 nm using a multimode microplate reader (EnVision 2104, PerkinElmer, Waltham, MA, USA). H_2_O_2_ levels were normalized to the total protein content. The normalized data are expressed as fold change relative to the control group.

### 4.12. Determination of ATP Content

An ATP content kit was utilized to measure the cellular ATP content through the manufacturer’s protocol (Beyotime, Shanghai, China, S0027). The luminescence intensity was recorded with a luminometer (EnVision 2104). Protein content was set as a reference to normalize the ATP levels. The data were expressed as relative ATP levels compared with the control.

### 4.13. Immunofluorescence Staining

A total of 4% (*v*/*v*) formaldehyde was employed to fix the fully differentiated C2C12 myotubes at room temperature for 20 min. A total of 0.1% Triton X-100 (Sigma-Aldrich, St. Louis, MO, USA) in PBS at room temperature for 1 h was used to permeabilize and block the fixed cells, after being washed with PBS twice. The cells were then incubated with myosin heavy chain antibody (MYHC, Abcam, Cambridge, UK, ab50967) at 4 °C overnight. The goat antimouse secondary antibody tagged with the Alexa Fluor 647 (Thermo Fisher Scientific, Waltham, MA, USA) was used to block the cells for 2 h at room temperature. Nuclei were counterstained with Hoechst 33342 (Thermo Fisher Scientific, Waltham, MA, USA) (10 μg/mL) for 20 min in the dark at 37 °C. After extensive washing, cell images were acquired using a confocal fluorescence microscope (Leica TCS SP8 SR, Wetzlar, Germany) and analyzed with ImageJ software (NIH, USA).

### 4.14. Label-Free LC-MS/MS Detection and Data Analysis

Lysis buffer (containing 8 M Urea and 100 mM TEAB, adjusted to pH 8.5) was used to extract proteins from the collected samples. Following centrifugation at 16,000× *g* for 10 min at 4 °C, the obtained supernatant was treated with 10 mM DTT for reduction and then alkylated with an adequate amount of IAA under dark conditions to avoid photodegradation. Subsequently, the samples were precipitated with acetone at −20 °C, followed by another round of centrifugation at 16,000× *g* for 10 min at 4 °C. The resulting pellet was re-dissolved in 100 mM TEAB (pH 8.5), and trypsin was added at a ratio of 100 μg protein to 2.5 μg trypsin. After thorough mixing, the samples were incubated at 37 °C for 18 h to allow complete enzymatic digestion. The peptide-containing supernatant was desalted using an Oasis HLB Extraction Cartridge (Waters, Milford, MA, USA). The desalted peptide solutions were collected and dried by a centrifugal vacuum evaporator at 60 °C. Finally, the dried samples were re-dissolved and subjected to LC-MS/MS analysis using an Ultimate 3000 RSLC nano system coupled with an Orbitrap Fusion Lumos Tribrid Mass Spectrometer (Thermo Fisher Scientific, Waltham, MA, USA), and the raw mass spectrometry data were searched and analyzed using Thermo Proteome Discoverer 2.4 software.

### 4.15. Western Blotting Analysis

The cell lysates were prepared with the RIPA lysis buffer (Applygen, C1053, Beijing, China) with protease inhibitors (Beyotime Biotechnology, P1045, Shanghai, China). The supernatant was collected after centrifugation at 14,000× *g* for 30 min at 4 °C, and the content of soluble protein in the supernatant was measured by a BCA protein assay kit (Beyotime Biotechnology, P0012S, Shanghai, China). Later, 30 μg protein was separated by 10% sodium dodecyl sulfate–polyacrylamide gel electrophoresis and transferred to polyvinylidene fluoride membranes. After being blocked with 5% nonfat milk dissolved by Tris-buffered saline with Tween 20 buffer for 1 h at room temperature, the membrane was blocked and incubated overnight at 4 °C with the following primary antibodies: PGC1α (Abclonal, Wuhan, China, A20995, 1:1000), SIRT1 (Cell Signaling Technology, Danvers, MA, USA, 9475, 1:250), anti-P-AMPK (Cell Signaling Technology, Danvers, MA, USA, 2535, 1:1000), anti-AMPK (Cell Signaling Technology, Danvers, MA, USA, 5832, 1:1000), and β-Actin (Abclonal, Wuhan, China, AC028, 1:4000). After being washed with Tris-buffered saline with Tween 20, the membrane was incubated with the secondary antibody for 3 h at room temperature. The blotting signals were developed with the chemiluminescence agent (Millipore, Burlington, MA, USA).

### 4.16. Statistical Data Analysis

Image software (version 1.53t, Java 1.8.0) was used for image processing and statistical analysis, while quantitative data were expressed as mean ± SEM based on at least three independent experiments analyzed and visualized using GraphPad Prism v10.0 (GraphPad Software, San Diego, CA, USA). The significance of differences between groups was assessed by one-way ANOVA followed by Tukey’s post hoc test, and *p* < 0.05 was considered statistically significant. All experiments were conducted with at least three independent biological replicates (*n* ≥ 3).

## 5. Conclusions

In summary, this study demonstrates that sporoderm disruption fundamentally remodels the structural characteristics of GLSP, resulting in enhanced cytoprotective activity. Through integrated structural characterization, proteomic profiling, mitochondrial functional assays, and signaling validation, we identify mitochondrial homeostasis as a central mechanism underlying GLSP-SB bioactivity. Activation of the SIRT1/AMPK–PGC1α axis enables preservation of mitochondrial integrity and energy metabolism under oxidative stress conditions. These findings not only provide mechanistic insight into the health benefits of *Ganoderma* spores but also highlight the importance of processing strategies in maximizing the functional potential of natural polysaccharides.

## Figures and Tables

**Table 1:** Compositional characteristics of GLSP-I and GLSP-SB.

Samples	Total Sugar Content (%)	Uronic Acid Content (%)	Residual Protein Content (%)
GLSP-I	65.9 ± 2.2	35.7 ± 1.3	3.7 ± 0.2
GLSP-SB	81.6 ± 3.6	18.5 ± 0.7	2.2 ± 0.2

**Table 2:** Molecular weight distribution of GLSP-I and GLSP-SB.

Samples	Retention Times (min)	Number Average Molecular Weight (Da)	Weight-Average Molecular Weight (Da)
GLSP-I	16.6	5,493,173	6,008,979
	21.0	311,564	466,017
	24.1	55,076	57,087
	27.0	8507	9297
	29.3	1398	1570
GLSP-SB	21.0	309,853	429,024
	24.5	45,921	46,505
	27.9	4989	5155

**Table 3:** Monosaccharide composition of GLSP-I and GLSP-SB.

Monosaccharides	GLSP-I	GLSP-SB
Fuc (%)	-	1.1
Ara (%)	17.8	46.6
Rha (%)	3.5	3.1
Gal (%)	9.2	16.0
Glc (%)	36.1	10.2
Xyl (%)	-	1.0
Man (%)	-	2.2
Gal-UA (%)	33.4	19.8

## Data Availability

The original contributions presented in this study are included in the article/Supplementary Materials. Further inquiries can be directed to the corresponding authors.

## Supplementary Materials

The following supporting information can be downloaded at: https://www.mdpi.com/article/10.3390/ijms27114741/s1.

## Abbreviations

The following abbreviations are used in this manuscript:

Akt	Protein kinase B
AMPK	AMP-activated protein kinase
ATP	Adenosine triphosphate
BCA	Bicinchoninic acid
CCK-8	Cell Counting Kit-8
CNS	Central nervous system
FT-IR	Fourier-transform infrared
Da	Dalton
DAPI	4′,6-Diamidino-2-phenylindole
DCFH-DA	2′,7′-Dichlorodihydrofluorescein diacetate
DEPs	Differentially expressed proteins
DMEM	Tert-butyl hydroperoxide
ROS	Reactive oxygen species
DMEM	Dulbecco’s Modified Eagle Medium
DMSO	Dimethyl sulfoxide
DTT	Dithiothreitol
FAD	Familial Alzheimer’s disease mutations
FBS	Fetal bovine serum
GLS	*Ganoderma lucidum* spores
GLSP	*Ganoderma lucidum* spore polysaccharides
GLSP-I	GLSP from intact spores
GLSP-SB	GLSP from sporoderm-broken spores
GO	Gene Ontology
GPC	Gel permeation chromatography
IAA	Iodoacetamide
LC-MS/MS	Liquid chromatography–tandem mass spectrometry
MAPK	Mitogen-activated protein kinase
Mw	Weight-average molecular weight
MYHC	Myosin heavy chain
Nrf2	Nuclear factor erythroid 2-related factor 2
P/S	Penicillin/streptomycin
PEG	Polyethylene glycol
PGC1α	Peroxisome proliferator-activated receptor gamma coactivator 1-alpha
PI3K/AKT/mTOR	Phosphoinositide 3-kinase/Protein kinase B/Mammalian target of rapamycin
RIPA	Radioimmunoprecipitation assay
SDS-PAGE	Sodium dodecyl sulfate-polyacrylamide gel electrophoresis
SEM	Scanning electron microscopy
SIRT1	Sirtuin 1
TBHP	Tert-butyl hydroperoxide
TFA	Trifluoroacetic acid
